# Identification of differentially expressed ovarian genes during primary and early secondary oocyte growth in coho salmon, *Oncorhynchus kisutch*

**DOI:** 10.1186/1477-7827-6-2

**Published:** 2008-01-18

**Authors:** John A Luckenbach, Dimitar B Iliev, Frederick W Goetz, Penny Swanson

**Affiliations:** 1 School of Aquatic and Fishery Sciences, University of Washington, Seattle, Washington 98195, USA; 2 Northwest Fisheries Science Center, National Oceanic and Atmospheric Administration-National Marine Fisheries Service, Seattle, Washington 98112, USA; 3 Great Lakes WATER Institute, University of Wisconsin, Milwaukee, Wisconsin 53204, USA; 4 Center of Reproductive Biology, Washington State University, Pullman, Washington 98164, USA

## Abstract

**Background:**

The aim of this study was to identify differentially expressed ovarian genes during primary and early secondary oocyte growth in coho salmon, a semelparous teleost that exhibits synchronous follicle development.

**Methods:**

Reciprocal suppression subtractive hybridization (SSH) libraries were generated from ovaries with perinucleolus (P) or cortical alveolus (CA) stage follicles and selected genes were assessed with quantitative PCR (qPCR). An assessment of changes in RNA composition during oocyte growth and its relationship to transcript levels was also conducted.

**Results:**

SSH revealed several differentially expressed genes during early oogenesis, some which will not likely be utilized until 1–3 years later in salmon. Zona pellucida glycoprotein (zp) genes, vitellogenin receptor (vldlr) isoforms, cathepsin B (ctsba), cyclin E (ccne), a DnaJ transcript (dnaja2), and a ferritin subunit (fth3) were significantly elevated at the P stage, while a C-type lectin, retinol dehydrogenase (rdh1), and a coatomer protein subunit (cope) were upregulated at the CA stage. Putative follicle cell transcripts such as anti-Müllerian hormone (amh), lipoprotein lipase (lpl), apolipoprotein E (apoe), gonadal soma-derived growth factor (gsdf) and follicle-stimulating hormone receptor (fshr) also increased significantly at the CA stage. The analysis of RNA composition during oocyte growth showed that the total RNA yield and proportion of messenger RNA relative to non-polyadenylated RNAs declined as oogenesis progressed. This influenced apparent transcript levels depending on the type of RNA template used and normalization method.

**Conclusion:**

In coho salmon, which exhibit a dramatic change in oocyte size and RNA composition during oogenesis, use of messenger RNA as template and normalization of qPCR data to a housekeeping gene, ef1a, yielded results that best reflected transcript abundance within the ovarian follicle. Synthesis of zp transcripts and proteins involved in yolk incorporation and processing occurred during primary growth, while increased expression of a CA component and genes related to lipid incorporation occurred concomitant with the appearance of CA, but prior to lipid accumulation. Significant increases in transcripts for fshr, gsdf, and amh at the CA stage suggest a role of FSH and TGFβ peptides in previtellogenic oocyte growth and puberty onset in female salmon.

## Background

Oocyte growth is a period of intense RNA synthesis, replication and redistribution of cytoplasmic organelles, and nutrient incorporation in oviparous vertebrates. In teleost fish, this period may encompass a significant portion of the lifespan, lasting well over a decade in some species. Despite this, research on fish oogenesis has primarily focused on vitellogenesis, final maturation and ovulation, while stages of primary and early secondary oocyte growth remain largely unexplored [1,2]. For example, it remains unclear what endocrine and/or intraovarian factors regulate oocyte growth and how this period may influence timing of puberty, fecundity, egg quality, and early embryogenesis.

Similar to primordial follicle development in mammals, primary oocyte growth in fish begins with the onset of meiosis and subsequent meiotic arrest in the diplotene stage of the first prophase. The oocytes are then completely enveloped by a monolayer of presumptive granulosa cells and a thin theca cell layer and epithelial sheath are added to the surface, forming the basic follicle structure [2,3]. As the follicle develops, the nucleus of the oocyte increases in size and numerous ribosome-producing nucleoli appear around its periphery ("perinucleolus" stage). Intense RNA synthesis occurs over this period and much of the RNA present in the fully grown oocyte is thought to be synthesized at this time [4-6]. During primary growth alone in fish, the oocyte volume may increase as much as 1,000- to 5,000-fold [1].

Initiation of secondary growth is signified by the appearance and accumulation of cortical alveoli (formerly yolk vesicles). These endogenously synthesized secretory vesicles, analogous to cortical granules in invertebrates and other vertebrates, are derived from Golgi bodies and play important roles in the fertilization response and early embryogenesis [7]. Upon fertilization, cortical alveoli fuse with the oocyte membrane and discharge their glycoprotein contents into the perivitelline space to prevent polyspermy and entry of microbes or pathogens. Cortical alveoli increase in number during early secondary growth, initially forming a ring around the periphery of the oocyte and then accumulating inward to the nucleus. In most fishes, a brief period of oocyte lipid deposition (lipid droplet stage) occurs late in the cortical alveolus stage and prior to significant yolk incorporation. Vitellogenesis (yolk incorporation) marks the final phase of secondary growth, during which dramatic follicle growth occurs as the oocyte sequesters vitellogenin, a hepatically derived yolk protein precursor, from the bloodstream [2].

Through recent large-scale genomic studies mainly conducted on zebrafish, salmonids, and *Fugu *pufferfish [8-13], a number of ovarian genes have been sequenced and catalogued in databases making it possible to identify many fish mRNAs, profile their expression, and determine their function(s). Genes involved in sex differentiation and early gametogenesis [14-16], and final oocyte maturation [17] have received considerable attention, while other studies have focused on specific gene families such as TGFβ superfamily members [18], zona pellucida glycoproteins [19], and vitellogenin receptor (very low density lipoprotein receptor, *vldlr*) [20]. Through these studies highly expressed ovarian genes, such as zona pellucida glycoprotein (*zp*) genes and egg lectins have been revealed. However, relatively few ovarian genes have been profiled in fish and little is known about temporal gene expression during oocyte growth.

The objective of this study was to identify differentially expressed genes during primary and early secondary oocyte growth with the ultimate goal of establishing what regulates these stages of oogenesis and identifying the intraovarian factors that drive (or block) puberty onset. Coho salmon, *Oncorhynchus kisutch*, was selected as a model because it is a semelparous species (spawns only once in its life and then dies) that exhibits synchronous follicle development. This unique reproductive life history allows for stage specific transcript analysis of a homogeneous clutch of follicles, which is not possible in iteroparous species like rainbow trout (*O. mykiss*) and Atlantic salmon (*Salmo salar*). Salmonids are also one of the best studied groups of fish and the large available repository of trout and Atlantic salmon ESTs facilitates identification and functional annotation of coho salmon transcripts. In this study, suppression subtractive hybridization (SSH) was conducted with ovaries containing follicles in primary or early secondary growth and putatively regulated genes identified by SSH were screened and then validated with real-time quantitative PCR (qPCR). Quantitative PCRs were developed for 17 genes identified by SSH and additional assays were developed for candidate genes of interest including known follicle cell transcripts, such as follicle-stimulating hormone receptor (*fshr*) and anti-Müllerian hormone (*amh*). Lastly, a thorough assessment of changes in the mRNA/total RNA ratio during oocyte growth was conducted to determine how gene expression results are influenced by rapid growth of follicles during oogenesis. This is germane to ovarian transcript analyses in other species and potentially other rapidly growing and/or differentiating tissues.

## Methods

### Animals and sampling

**Figure 1:**
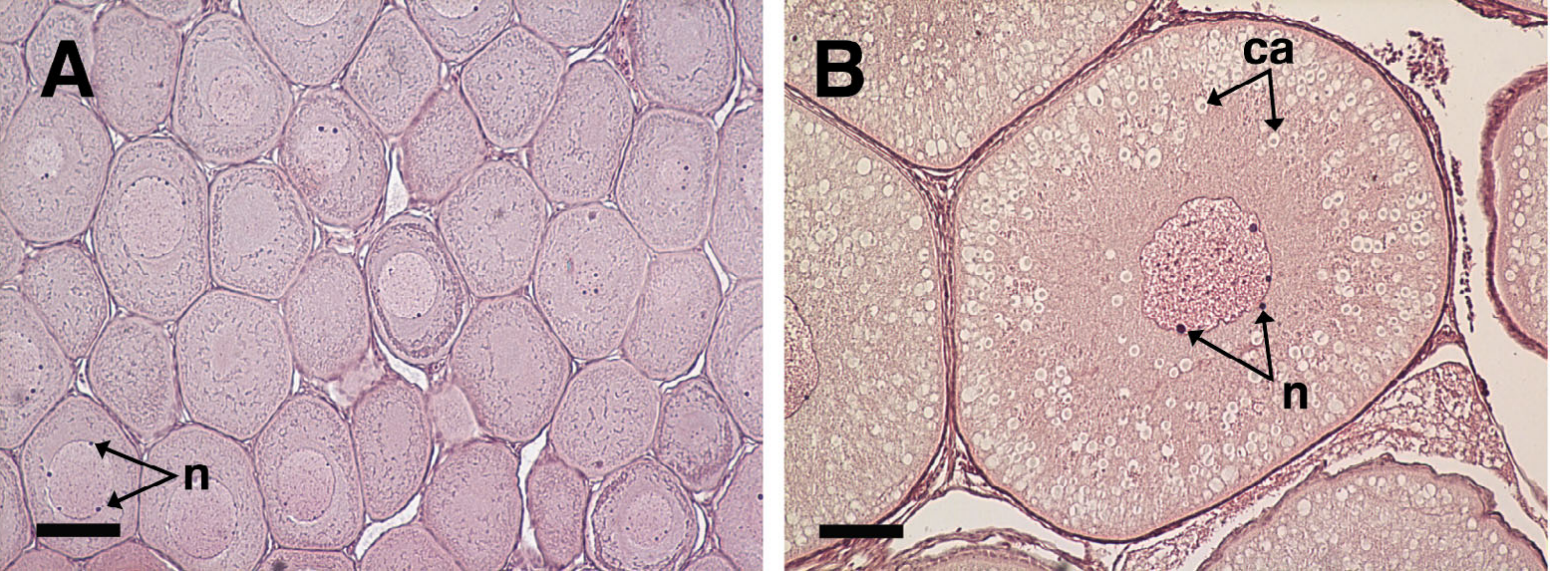
**Histological sections of coho salmon ovaries with perinucleolus (A) and mid-cortical alveolus stage follicles (B)**. Panel A shows a representative ovary from cohort 1 fish and panel B shows a representative ovary from cohort 2 fish used for subtractive hybridization and qPCR validations. The scale bar = 100 μm in each panel; n, nucleoli; ca, cortical alveoli.

Coho salmon (2004 and 2003 brood) were reared at the Northwest Fisheries Science Center (Seattle, WA) in recirculated fresh water under a simulated natural photoperiod and fed a standard ration of a commercial diet. These salmon typically spawn in December at 3 years of age and the same cohorts (2004 brood = cohort 1, 2003 brood = cohort 2) were used for all experiments. In October 2005, female salmon (N = 43 fish of cohort 1; N = 20 fish of cohort 2) were euthanized and their ovaries removed and weighed. At this time, cohort 1 fish were 0^+ ^age (10 months old), 72–103 mm fork length (FL) and 4.5–13.3 g body mass with an ovary mass of 0.029 ± 0.001 (mean ± SEM) and gonadosomatic index (GSI) of 0.32 ± 0.01. Cohort 2 fish were 1^+ ^age (22 months old), 194–235 mm FL and 93.5–157.8 g body mass with an ovary mass of 0.400 ± 0.021 g and GSI of 0.33 ± 0.01. A piece of ovary from each fish was fixed in Bouins for paraffin histology [21], and the remaining tissue was snap frozen for later RNA isolation. Histology revealed that in October 2005, ovaries of cohort 1 possessed primary growth follicles at the perinucleolus (P) stage containing minimal Balbiani material and no cortical alveoli (Fig. 1). Ovaries of cohort 2 contained follicles that were early to mid-cortical alveolus (CA) stage with cortical alveoli filling greater than 50% of the ooplasm. Samples were selected for SSH that represented primary growth (P stage, cohort 1) and early secondary oocyte growth (mid-CA stage, cohort 2). Salmon used for qPCR validation of the SSH results (N = 10 fish/stage) possessed ovaries that were in the same stages as those used for SSH. For the template RNA assessment (see below), fish were sampled as described above at a later time point in August 2006 (N = 5/cohort). At this time, fish from cohort 1 were 1^+ ^age (20 months old) and follicles were generally late P stage with most oocytes showing very few cortical alveoli in the periphery of the ooplasm, while cohort 2 fish were 2^+ ^age (30 months old) and oocytes were in the yolk granule stage.

Fish were reared and handled according to the policies and guidelines of the University of Washington Institutional Animal Care and Use Committee (IACUC Protocol #2313-09).

### RNA isolation and cDNA synthesis

Ovarian total RNA was isolated with Tri-Reagent (Molecular Research Center, Cincinnati, OH). For SSH, total RNA pools were generated from 18 P stage and 3 CA stage ovaries (whole ovaries minus the part for fixation) and mRNA was further isolated via the PolyATract mRNA Isolation System (Promega, Madison, WI). For individual samples for qPCR, the MicroPoly(A)Purist kit (Ambion, Austin, TX) was used. Samples for qPCR were reverse transcribed with SuperScript II (Invitrogen, Carlsbad, CA) and either 100 ng mRNA or 1000 ng total RNA in 20 μl reactions.

### Subtractive hybridization and analysis

SSH was performed with 1 μg of mRNA per stage using the PCR-Select cDNA Subtraction and Advantage cDNA PCR kits (Clontech, Palo Alto, CA). Subtracted cDNA samples were cloned in TOPO pCR 2.1 (Invitrogen) and plated on LB agar containing 100 μg/ml kanamycin. A total of 288 clones per library were randomly selected for sequencing with Big Dye Terminator (Applied Biosystems (ABI), Foster City, CA) on an ABI 3730 sequencer using the M13 reverse primer. Sequence chromatogram files were trimmed for quality (phred), vector-screened (cross match), and analyzed in the following manner to determine transcript identities: sequences ≤ 30 bp were removed and the remaining sequences were analyzed for redundancy with CAP3 [22], and analyzed locally using blastx against the NCBI nonredundant (nr) protein database, blastn against the NCBI nucleotide (nt) database, blastn against the EST database (est_others), and blastx against the Gene Ontology (GO) database [23]. The e-value cutoff was 10^-5 ^for blast searches. Nomenclature of the Zebrafish Information Network (ZFIN) was used where possible, except for the *zp *genes for which we followed the recommended nomenclature of Spargo and Hope [24].

### Quantitative PCR

Approximately 60 PCR primer sets were designed from sequences obtained by SSH with MacVector software (Accelrys, San Diego, CA) and undigested SSH cDNA was used as template to screen for differentially expressed genes with semi-quantitative PCR as described by Goetz et al. [25]. Genes that appeared to be differentially expressed in the initial screen were measured with real-time qPCR using primers listed in Table 1. Assays were run on an ABI 7700 Sequence Detector in 96-well plates using the standard cycling conditions: 50°C for 2 min, 95°C for 10 min, followed by 40 cycles of 95°C for 15 s and 60°C for 1 min. Reactions consisted of 1× Power SYBR Green PCR master mix (ABI), 150 nM of each gene-specific forward and reverse primer, and 0.01 ng cDNA template (based on the mRNA loaded into the RT reactions). For total RNA samples used in the template RNA assessment, 1 ng cDNA was loaded per reaction.

**Table 1:** Primer sequences for qPCR.

Putative gene name and symbol	5'-Forward primer-3'	5'-Reverse primer-3'	Product size (bp)
*Genes identified by SSH*			
Factor in the germline α (*figla*)	TCTTGAAGAATGCGATCAACTATGC	CACTCACGTCTCCATCTCCTGC	177
Zona pellucida protein X (*zpx*)	TTGTGACACCTTGTTCCGCC	TCGCAACTACACGCCCTTAGAC	257
Zona pellucida protein C (*zpc*)	CAGGAAAGCATCCACAGTGAAGC	TTTGTTACGACATCCAATGACCC	153
Egg envelope glycoprotein (*eeg*)	TGGTGTCATCTGTCTCTTGAGGC	TTTGAAGCAGTTCTACGGCTCG	233
Somatic lipoprotein receptor (*vldlro*)	AACAGAGCAACAACCAGATGCC	TAGAACCAGTATCACGCCCTGC	278
Cathepsin B, a (*ctsba*)	GAACACTGACTGGGGAGATAATGG	CTCTGATAGGGTGGAGTTTCCTTTC	222
Cathepsin D (*ctsd*)	CGTCATCTTTGACTCCCGATCC	GCACAAGTTTCCATTTGCTTTTCTG	175
Cathepsin Z (*ctsz*)	TCCCATCGTTCCCAAAACCTAC	TGTTCCCAAGGCAAAGCACG	200
Serum lectin isoform 2 (*lcal*)	CAGGTTCTTATGGAGCACACGG	GTTCAAGCAAAGGTGGAAAAGGAC	203
Rhamnose binding lectin STL3 (*lrham*)	ACCAACTCACCGACACCAACTG	CCCAATGGAACACACATCGTG	290
Alveolin (*alv*)	ACAGAGAAATCACCTGAGCCCC	GGAGAAATAAAACCACTGCCTGC	253
Ferritin H-3 (*fth3*)	ACTCTCCTTCCAGAACAAGCGAG	TTGGTGAGGTTGGTGATGTGG	264
DnaJ subfamily A member 2 (*dnaja2*)	TGTCTGCCATCTAAGTGTTTCAACG	CGCTGTAAAAAGTGCGAGGGC	278
Cyclin E (*ccne*)	GTTCAAAGGAATCCCAGCAGATG	TCACCGTAGCACAATAAACACCC	215
Retinol dehydrogenase 1 (*rhd1*)	GTTCAGGCAGATTCACCACCC	CGATGACCCCGTTCAGGTTC	210
Coatomer complex protein, epsilon (*cope*)	CAGATGTCTCCATTGTCTCAAGCAG	TGACCCAGCACTTAGGCAAAGC	175
Anterior pharynx defective 1b (*aph1b*)	CTGCTGAAGAAGGCAAACGAAG	AGGAATCAAAGAACACCACACCC	268
			
*Candidate genes*			
Vitellogenin receptor (*vldlr*)	TCTGGAAGTGTGATGGAGAGAAGG	TCGCTGGGTGAACATTTAGCC	337
Apolipoprotein E (*apoe*)	AGGGAGGACATCCAGACCAAGTTG	CGTTTCTTCAGTTTGCGGGTGTAG	218
Lipoprotein lipase (*lpl*)	GTGCCTCAACTGCCGTAAGAAC	TGCCCCAAATGTCAGACCAG	365
FSH receptor (*fshr*)	GACGCACATCAGAGTGTTTCCC	GTAGAACCCTCAGTCCAGTGTTGC	242
Anti-Müllerian hormone (*amh*)	TCACTTTCACCAGTCACTCTCTGC	CACTTCTTGTTCCGTCACCAATC	204
Gonadal soma-derived growth factor (*gsdf*)	CCAGAATCAAGAAGGAATACGCAG	TGGGAAAAGAGAGGAGAGCAGG	317
Elongation factor-1α (*ef1a*)	CCCCTGGACACAGAGATTTCATC	AGAGTCACACCGTTGGCGTTAC	409

Triplicate standard curve samples generated from a serial dilution of pooled RNA from ovaries of 6 previtellogenic coho salmon were included in each plate. The standard curve (log input cDNA template versus cycle threshold) was linear for cDNA template ranging from 0.001–1 ng. Results were analyzed using the relative standard curve method (for details see the ABI Prism 7700 Sequence Detection System User Bulletin #2, P/N 4303859). Experimental samples were run in duplicate for the housekeeping gene (see below) and not replicated for the target genes. No amplification controls (NAC) that lacked reverse transcriptase and a no template control (NTC) that lacked template altogether were evaluated in each plate to confirm the absence of genomic DNA in the RNA preparations and the absence of PCR carryover contamination, respectively. Negative control samples showed either no detectability or negligible values (>10 Ct separation from samples). Melt curve analysis was included for each target gene to ensure that a single product was amplified. In cases where more than one product was generated the PCR was redesigned. In addition, a qPCR product from each plate was directly sequenced to verify that the target was successfully amplified. Target gene results were normalized to elongation factor-1 alpha (*ef1a*), which served as a housekeeping gene and has been used in previous gonadal studies [e.g., [15,26]]. *Ef1a *transcript levels were not different across stages when mRNA was used as template (P = 0.5). The lowest normalized value for each gene was arbitrarily set to 1 to enhance data presentation.

### Template RNA assessment

The RNA composition and size of ovarian follicles change dramatically during oocyte growth, both of which can influence transcript levels depending on the method of normalization. Therefore, we compared transcript levels when measured from total RNA versus mRNA as template, correcting for follicle number and RNA recovery. Follicles were counted prior to RNA isolation and the expression of several genes was measured in total RNA and mRNA preparations from the same individual ovary samples. Briefly, fish were euthanized and body mass, FL, and ovary mass recorded. Fragments weighing ~40 mg taken from the middle region of the ovary were snap frozen for RNA analysis and an additional piece was fixed in Bouins for histology. Triplicate fragments of fresh ovary were weighed and the follicles counted under a dissecting microscope to determine the average follicle mass for each fish. Fecundity and RNA yield per follicle estimates were based on average follicle mass data. Gene expression was assessed in total RNA and mRNA preparations for a housekeeping gene, *ef1a*; a downregulated gene, ferritin H-3 (*fth3*); and an upregulated gene, *fshr*.

### Statistical analysis

Gene expression results were compared by unpaired t-tests and Welch's correction was applied in cases where variances were unequal across stages (Prism 4, GraphPad Software, San Diego, CA). The minimum level of statistical significance was set at P < 0.05.

## Results

### General library statistics

The SSH library putatively enriched in cDNAs/transcripts that were more abundant at the P stage yielded 275 sequences (>30 bp) with an average size of 420 bp that clustered to form 55 contigs (of two or greater sequences with >90% identity) and 155 singletons. Approximately 80% of the sequences were identified as similar to annotated genes, while 15% had similarity to unannotated genes (entries of unknown function, e.g. hypothetical proteins) and 5% were novel (no significant identity to GenBank sequences). The SSH library putatively enriched in genes upregulated at the CA stage yielded 267 sequences with an average size of 401 bp, which clustered to form 47 contigs and 103 singletons. Approximately 71% of the sequences were similar to annotated genes, 16% were similar to unannotated genes, and 13% were novel. The two predominant putative genes in the P and CA libraries with a frequency of ~10% were *fth3 *and serum lectin isoform 2 (*lcal*), respectively.

Expressed sequence tags (ESTs) were submitted to the NCBI database [GenBank: EX152144–EX152685]. Genes further assessed with qPCR are listed in Table 2.

**Table 2:** Selected salmon ovarian genes revealed by SSH and further assessed with qPCR.

Putative gene identity	EST	Size of query (bp)	E-value	Species	Accession no.	GO molecular function term(s)
Factor in the germline alpha (*figla*)	EX152185	257	9E-14	*K. marmoratus*	ABG89136	GO:0030528 transcription regulator activity
Zona pellucida glycoprotein C (*zpc*)	EX152517	277	8E-09	*D. rerio*	AAI16532	
Zona pellucida protein X (*zpx*)	EX152510	429	9E-27	*S. aurata*	AAY21008	
Egg envelope glycoprotein (*eeg*)	EX152158	592	6E-34	*D. rerio*	XP_001335392	
Somatic lipoprotein receptor (*vldlro*)	EX152595	546	8E-14	*O. mykiss*	CAA05874	GO:0004872 receptor activity
						GO:0005509 calcium ion binding
Cathepsin B, a (*ctsba*)	EX152225	495	4E-29	*D. rerio*	NP_998501	GO:0004197 cysteine-type endopeptidase activity
Cathepsin D (*ctsd*)	EX152680	327	1E-123 *	*O. mykiss*	U90321	GO:0004190 aspartic-type endopeptidase activity
						GO:0004192 cathepsin D activity
						GO:0004194 pepsin A activity
						GO:0016787 hydrolase activity
Cathepsin Z (*ctsz*)†	EX152609	624	5E-172 *	*O. mykiss*	AAT94061	GO:0004197 cysteine-type endopeptidase activity
						GO:0008234 cysteine-type peptidase activity
Serum lectin isoform 2 (*lcal*)	EX152541	513	1E-34	*S. salar*	AAO43606	GO:0005529 sugar binding
Rhamnose binding lectin STL3 (*lrham*)	EX152294	324	8E-52	*O. mykiss*	BAA92257	GO:0005529 sugar binding
						GO:0016524 latrotoxin receptor activity
Alveolin (*alv*)	EX152194	532	4E-36	*O. latipes*	BAA90750	GO:0008237 metallopeptidase activity
						GO:0008533 astacin activity
Cyclin E (*ccne*)	EX152152	800	3E-53	*D. rerio*	NP_571070	GO:0016301 kinase activity
Ferritin H-3 (*fth3*)	EX152267	552	2E-91	*O. mykiss*	BAA13148	GO:0005488 binding
						GO:0005506 iron ion binding
						GO:0008199 ferric iron binding
						GO:0016491 oxidoreductase activity
						GO:0046872 metal ion binding
						GO:0046914 transition metal ion binding
DnaJ subfamily A member 2 (*dnaja2*)	EX152440	334	2E-55	*D. rerio*	NP_998658	GO:0031072 heat shock protein binding
						GO:0051082 unfolded protein binding
Coatomer protein epsilon subunit (*cope*)††	EX152380	438	6E-44	*O. mykiss*	AAM18476	
Anterior pharynx defective 1b (*aph1b*)	EX152190	601	6E-90	*D. rerio*	NP_956409	GO:0005515 protein binding
Retinol dehydrogenase 1 (*rdh1*)	EX152534	565	7E-71	*O. latipes*	ABQ09278	GO:0016491 oxidoreductase activity

† The top blast hit was cathepsin Y, which is a synonym for cathepsin Z used here.

†† The top blast hit was named VHSV induced protein, but this appears to be a coatomer protein subunit based on similarity to other sequences.

* The blastn similarity score is shown instead of blastx due to the sequence corresponding primarily or completely to the 3' UTR.

Complementary DNA sequences for additional candidate genes were obtained from a follicle/interstitial cell enriched library [29].

### Gene expression analysis

**Figure 2:**
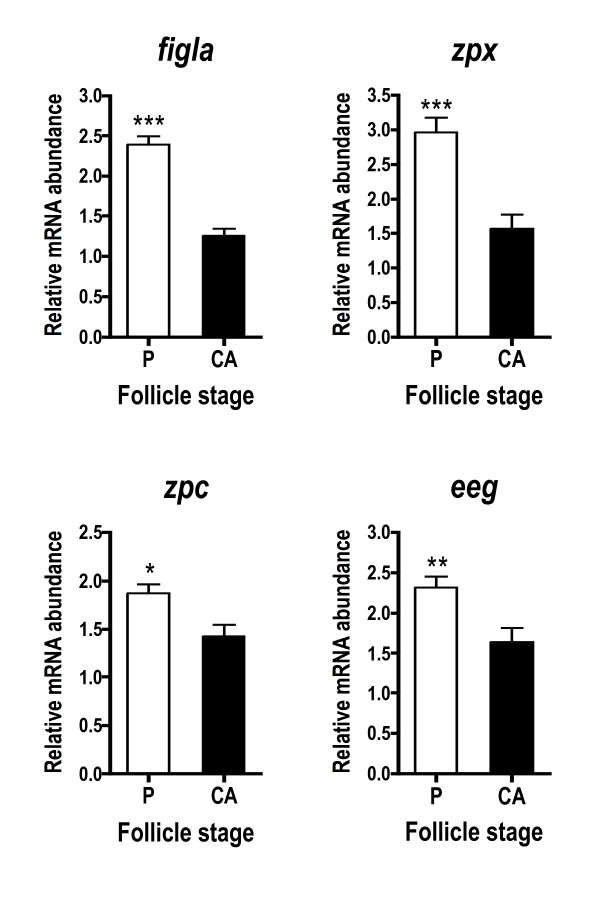
**Abundance of zona pellucida glycoprotein-related transcripts during primary and early secondary oocyte growth in salmon**. Open bars denote perinucleolus (P) stage samples, while solid bars denote cortical alveolus (CA) stage samples. Each bar represents the mean + SEM of 10 fish per stage with *P < 0.05, **P < 0.01 and ***P < 0.001 indicating statistical differences. Relative transcript levels were normalized to *ef1a*.

**Figure 3:**
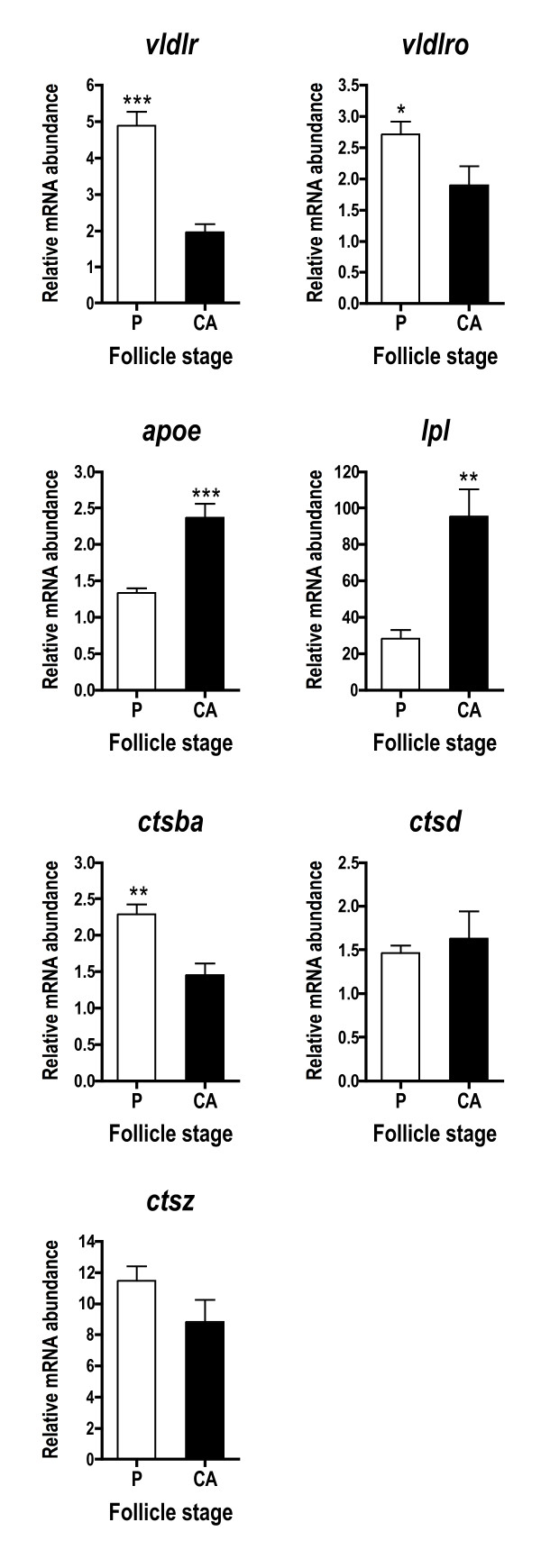
**Abundance of lipoprotein/lipid uptake and processing transcripts during primary and early secondary oocyte growth**. Open bars denote perinucleolus (P) stage samples, while solid bars denote cortical alveolus (CA) stage samples. Each bar represents the mean + SEM of 10 fish per stage with *P < 0.05, **P < 0.01 and ***P < 0.001 indicating statistical differences. Relative transcript levels were normalized to *ef1a*.

Zona pellucida genes, including an EST similar to factor in the germline alpha (*figla*), were more abundant in P stage follicles compared to CA stage follicles (Fig. 2). All genes falling into this cascade (*figla*, *zpx*, *zpc*, *eeg*) showed a similar pattern, but the fold-difference across stages was most pronounced for *figla *and *zpx *where mRNA levels were elevated ~2-fold at the P stage. Several genes associated with lipoprotein uptake and yolk processing exhibited this pattern, including *vldlr*, somatic lipoprotein receptor (v*ldlro*), and cathepsin B (*ctsba*) (Fig. 3). Cathepsin D (*ctsd*) and cathepsin Z (*ctsz*), however, were not differentially expressed across stages when assessed with qPCR (Fig. 3). Lipoprotein lipase (*lpl*) and apolipoprotein E (*apoe*) mRNA levels were significantly higher during the CA stage.

**Figure 4:**
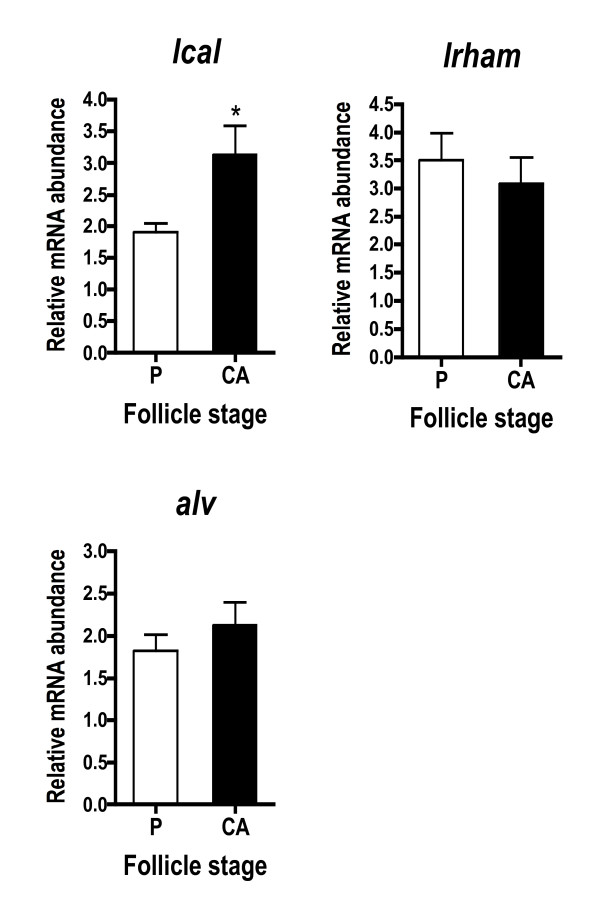
**Abundance of cortical alveoli component transcripts during primary and early secondary oocyte growth**. Open bars denote perinucleolus (P) stage samples, while solid bars denote cortical alveolus (CA) stage samples. Each bar represents the mean + SEM of 10 fish per stage with *P < 0.05, **P < 0.01 and ***P < 0.001 indicating statistical differences. Relative transcript levels were normalized to *ef1a*.

Genes encoding cortical alveoli components had variable expression patterns (Fig. 4). *Lcal *was significantly elevated in CA stage samples, whereas rhamnose binding lectin STL3 (*lrham*) and alveolin (*alv*) were not statistically different across stages.

**Figure 5:**
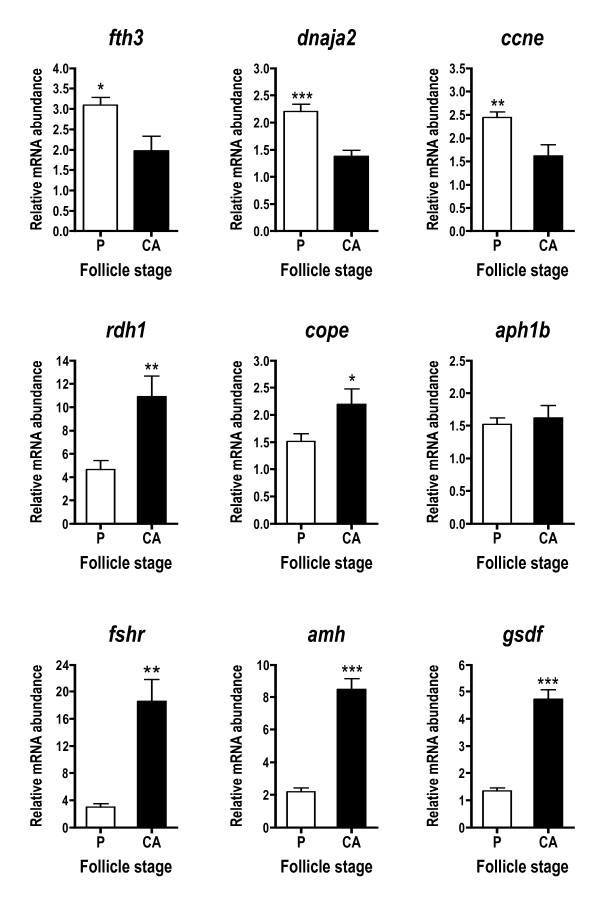
**Abundance of other ovarian transcripts during primary and early secondary oocyte growth**. These include likely follicle cell transcripts. Open bars denote perinucleolus (P) stage samples, while solid bars denote cortical alveolus (CA) stage samples. Each bar represents the mean + SEM of 10 fish per stage with *P < 0.05, **P < 0.01 and ***P < 0.001 indicating statistical differences. Relative transcript levels were normalized to *ef1a*.

A number of other genes not falling into the above cascades were differentially expressed across stages (Fig. 5). *Fth3*, DnaJ subfamily A member 2 (*dnaja2*), and cyclin E (*ccne*) transcripts were significantly elevated at the P stage, while retinol dehydrogenase 1 (*rdh1*) and coatomer protein epsilon subunit (*cope*), showed the opposite pattern and were higher at the CA stage. Anterior pharynx defective 1b (*aph1b*) was not differentially expressed based on qPCR results. Finally, three transcripts likely originating from the follicle cells, *fshr*, *amh*, and gonadal soma-derived growth factor (*gsdf*) were dramatically upregulated at the CA stage.

### Template RNA assessment

The mean quantity of total RNA isolated per gram of ovary for cohorts 1 and 2 were 4750 and 1509 μg, respectively, and thus the total RNA yield was about 3 times greater per g ovary for cohort 1 (Table 3). However, because there were 40 times more follicles per tissue mass for cohort 1, the total RNA yielded per follicle was lower relative to cohort 2. The average mRNA yielded per μg total RNA was also greater for cohort 1 relative to cohort 2. Again, due to the difference in follicle number the mRNA yielded per follicle was actually lower for cohort 1. Based on mRNA yields, total RNA from cohort 1 and 2 consisted of ~12% and 2% mRNA, respectively.

**Table 3:** Analysis of total and messenger RNA yields and transcript levels across different stage ovaries of coho salmon (N = 5 fish/stage) in August 2006.

Parameter	Cohort 1	Cohort 2	Cohort 1: mRNA based	Cohort 2: mRNA based	Fold change (mRNA)	Cohort 1: total RNA based	Cohort 2: total RNA based	Fold change (total RNA)
Body mass (g)	77.1 ± 9.5	736.5 ± 33.8						
Fork length (cm)	17.7 ± 0.7	36.4 ± 0.9						
Paired ovary mass (g)	0.22 ± 0.02	8.51 ± 0.71						
Gonadosomatic Index (GSI)	0.30 ± 0.03	1.15 ± 0.05						
Follicle stage	late perinucleolus	yolk granule						
Est. fecundity (no. of follicles)	3361 ± 192	2999 ± 462						
μg total RNA/g ovary	4750 ± 80	1509 ± 40						
μg total RNA/follicle	0.31 ± 0.01	4.50 ± 0.50						
ng mRNA/μg total RNA	117.8 ± 4.0	23.4 ± 3.2						
ng mRNA/follicle	36.4 ± 1.6	99.5 ± 4.7						
% mRNA	11.8 ± 0.4	2.3 ± 0.3						
								
*Housekeeping gene*								
*ef1a *per template RNA			1.02 ± 0.01	1.18 ± 0.03	*up 1.2*	1.94 ± 0.05	1.33 ± 0.09	*down 1.5*
*ef1a *per follicle			1.14 ± 0.04	1.16 ± 0.04	*nc*	5.61 ± 0.15	1.24 ± 0.08	*down 4.5*
								
*Downregulated gene*								
*fth3 *per template RNA			4.25 ± 0.20	1.35 ± 0.13	*down 3.2*	18.60 ± 0.46	1.69 ± 0.26	*down 11.0*
*fth3 *per follicle			4.89 ± 0.20	1.38 ± 0.16	*down 3.5*	58.27 ± 1.99	1.69 ± 0.22	*down 34.5*
*fth3*/*ef1a*			4.69 ± 0.21	1.29 ± 0.13	*down 3.6*	9.81 ± 0.25	1.29 ± 0.19	*down 7.6*
								
*Upregulated gene*								
*fshr *per template RNA			1.58 ± 0.35	12.48 ± 2.53	*up 7.9*	1.16 ± 0.05	3.44 ± 0.38	*up 3.0*
*fshr *per follicle			1.82 ± 0.43	12.57 ± 2.70	*up 6.9*	1.22 ± 0.06	1.17 ± 0.12	*nc*
*fshr*/*ef1a*			1.57 ± 0.35	10.81 ± 2.41	*up 6.9*	1.23 ± 0.08	5.25 ± 0.34	*up 4.3*

Cohort 1 fish were brood year 2004 and cohort 2 fish were brood year 2003.

nc, no change

*Ef1a *transcript levels were similar across stages based on template mRNA (0 to 1.2-fold change), but decreased (1.5 to 4.5-fold) in more advanced ovaries based on total RNA template (Table 3). This decrease was most pronounced when total RNA based data were expressed on a per follicle basis. Other housekeeping genes, such as acidic ribosomal protein, showed results similar to *ef1a *(data not shown). F*th3 *declined in more advanced ovaries with all means of analysis, but this decline was most pronounced with total RNA template. F*shr *showed a 6.9 to 7.9-fold increase with mRNA template, while total RNA based samples showed either no increase or a diminished increase (0 to 4.3-fold increase). Results were most skewed in total RNA preparations when presented on a per follicle basis and most closely resembled mRNA preparations when normalized to *ef1a*.

## Discussion

The goal of this study was to identify differentially expressed ovarian genes during the little studied periods of primary and early secondary oocyte growth in coho salmon. A number of differentially expressed genes were successfully identified through SSH and qPCR that are known to play important roles during oogenesis, such as zona pellucida development, sequestration and processing of lipoproteins, cell cycle control, and the fertilization response. Interestingly, some genes involved in vitellogenesis, fertilization, and embryogenesis were more highly expressed during primary growth than early secondary growth. This pattern is intriguing because proteins encoded by many of these genes are not thought to be utilized until 1–3 years later in this species. Through work mainly conducted in *Xenopus *it has been well documented that maternal RNAs are deposited in the oocyte early in oogenesis and then stored as messenger ribonucleoprotein particles (mRNPs), which contain proteins such as Y-box proteins and DEAD-box RNA helicases that mask RNA from the translational apparatus [5,27,28]. In the present study, the *ccne *transcript was upregulated during primary growth and would likely represent a maternal RNA based on its role in early embryogenesis (see below). However, other transcripts, such as *vldlr *and cathepsins, associated with later aspects of oogenesis itself were also highly expressed during primary growth. Although protein data are necessary to resolve this issue, one possible explanation for the early transcription of such genes is that some oogenesis related mRNAs are subject to masking during early oogenesis as described for traditional maternal RNAs.

Other transcripts such as *lpl*, *apoe*, *lcal*, *rdh1*, *amh*, *gsdf*, and *fshr *increased significantly during early secondary growth when CA are abundant in the oocytes, but lipid droplets are not yet evident. Some of these transcripts were likely derived from the follicle cells and showed a dramatic increase across stages (2 to 6 fold). These data together with results of previous studies discussed in detail below, suggest an increased role of the follicle cells during early secondary growth in coho salmon and potential regulation of this transition by FSH and TGFβ family peptides.

Based on GO annotation and examination of the literature, the majority of genes revealed by SSH were of oocyte origin. The SSH method employed enriches for rare transcripts through PCR steps; however it appears that copious mRNA from the oocytes overshadowed differentially expressed genes originating from the follicle/interstitial cells. This idea is supported by the observed differences in abundance of follicle cell transcripts, such as *amh *and *gsdf*, which were candidate genes obtained from a follicle/interstitial cell enriched library [29]. As first noted by Goetz and colleagues [30] these findings suggest that it is necessary to enrich for these cell layers prior to SSH to increase the likelihood of revealing rare transcripts of the granulosa or theca/interstitial cells.

### Analyzing transcript levels across stages of oogenesis

One problem with studying the ontogeny of ovarian gene expression, especially in oviparous vertebrates with large eggs, is the dramatic change in oocyte size and RNA composition that occurs during oogenesis. Changes in the size and number of follicles per tissue mass and instability of housekeeping genes complicate across stage comparisons making it difficult to interpret transcript abundance data in a biologically relevant way [e.g., [21,31-34]]. To gain insight into this problem, we quantified RNA yields per tissue mass and per follicle, and transcript abundance in total RNA and mRNA preparations. As oogenesis progressed, total RNA yielded per tissue mass and the proportion of mRNA relative to non-polyadenylated RNA declined (Table 3). Furthermore, the yield of RNA per follicle was higher in more advanced follicles but the amount of mRNA did not increase proportional to follicle size. This change in RNA composition had a significant effect on apparent transcript levels for a variety of genes expressed in the oocyte and follicle cells depending on whether mRNA or total RNA was used as template for cDNA synthesis. Housekeeping genes, such as *ef1a*, were similarly expressed across stages when mRNA was used as the template, irrespective of whether data were calculated per unit template RNA or per follicle. Our results indicate that if total RNA is used as a template without normalization to a housekeeping gene one can get misleading results, such as an apparent decline in transcripts that are actually stably expressed, an accentuated decline in downregulated genes, or no change in transcripts for upregulated genes. Results were most skewed in total RNA preparations when presented on a per follicle basis and most closely resembled mRNA based results when normalized to *ef1a*. In summary, use of mRNA as template and normalization of qPCR data to *ef1a *generated results that best reflected transcript abundance within the follicle.

### Zona pellucida glycoprotein genes

The zona pellucida is the acellular membrane that not only encloses the oocyte, but is also critical to optimal oocyte growth and preventing polyspermy after fertilization. The zona pellucida in vertebrates consists of highly sulfated zona pellucida glycoproteins (ZPs). Four ZP subfamilies, ZPA, ZPX, ZPB and ZPC, have been described and are each found in fish except ZPA [19,24]. Several coho salmon *zp *genes were revealed by SSH and all exhibited significantly elevated mRNA levels during primary growth relative to early secondary growth (Fig. 2). Consistent with this, other studies have shown that *zp *transcripts are abundant in oocytes during early oogenesis and make up a significant portion of the ovarian transcriptome [7-9,13,31].

Few studies in fish have focused on the *zp*-related transcription factor, *figla*. In mammals, however, *figla *is required for primordial follicle development and transcription of the *zp *genes [6,35]. Like the *zp *genes, *figla *is highly expressed in primary oocytes and has been localized to the ooplasm in medaka fish, *Oryzias latipes *[14]. In the present study, mRNA levels for both the *zp *genes and *figla *EST were higher during primary growth. The similar profile of *figla *and the *zp *genes in this study along with work in other species [35,36] suggests that transcription of this family of genes is highly coordinated.

### Lipoprotein uptake and processing

Several transcripts associated with vitellogenesis, such as *vldlr *and *vldlro*, were highly expressed during primary growth and declined significantly by early secondary growth (Fig. 3). Vldlr has been widely studied and is responsible for uptake of hepatically-derived vitellogenin by the oocyte [2]. On the other hand, very little is known about *vldlro*, which contains an *O*-linked sugar domain, is expressed in the ovary and somatic tissues of some fishes [33,37,38], and is thought to mediate uptake of lipoproteins other than vitellogenin [37]. Findings of the present study are consistent with work in rainbow trout which showed that transcription of *vldlr *began shortly after female differentiation [39] and declined by early vitellogenesis, becoming nearly undetectable by mid-vitellogenesis [20]. Given that *vldlr *mRNA levels are low during much of the period of active uptake of vitellogenin, it is widely believed that the Vldlr protein is recycled during vitellogenesis [20,37,40].

Two genes, *lpl *and *apoe*, associated with lipid or lipoprotein uptake by oocytes increased 2–3 fold during early secondary growth. Lpl cleaves fatty acids from plasma lipoproteins and thus facilitates lipid transport across biological membranes, while Apoe is a lipid binding protein that mediates recognition and internalization of plasma lipoproteins by cell surface receptors. The timing of this increase is interesting considering that the CA stage just precedes the appearance of lipid inclusions within the salmon oocyte. Few studies have focused on Lpl and Apoe in the teleost ovary. However, studies in rainbow trout [41,42] demonstrated that *lpl *mRNA levels and Lpl activity increased steadily during vitellogenesis and peaked at late vitellogenesis. In sea bass (*D. labrax*), *lpl *mRNA was localized to the follicle cells and high *lpl *mRNA levels and Lpl activity coincided with the appearance of oocyte lipid inclusions [43]. Thus mounting evidence suggests that Lpl may be involved in lipid uptake associated with secondary oocyte growth in fish. To our knowledge, *apoe *mRNA has been measured in the ovary in only one other study in fish [15], which demonstrated expression during very early oogenesis. Together these data indicate there is increased expression of genes associated with lipid transport prior to significant lipid incorporation.

Cathepsins are lysosomal enzymes that in the oocyte are responsible for proteolytically cleaving vitellogenin into its constituent yolk proteins and play a role in oocyte reabsorption during atresia [2,44,45]. Coho salmon cathepsins revealed by SSH showed different patterns of expression during oocyte growth. The *ctsb *transcript was more abundant during primary growth relative to early secondary growth. Both *ctsd *and *ctsz *mRNA levels were not different across stages and would be considered false positives of SSH, which are commonly encountered with this technique [46]. Ctsd is considered the major protease responsible for cleaving vitellogenin into yolk proteins [2]. However, in the barfin flounder, Ctsb was implicated in this role [47]. In the teleost, *Fundulus heteroclitus*, *ctsz *expression was relatively stable during oocyte growth and maturation [48], and in rainbow trout *ctsz *was upregulated during maturation [17] and correlated with egg quality [49]. Data generally suggest that ovarian cathepsins may be regulated post-transcriptionally and thus transcript abundance may not correlate well with cathepsin enzymatic activity [42,44,50].

### Cortical alveoli components

Studies in *Xenopus *have shown that 70% of the proteins contained in cortical granules are lectins [7]. In fish oocytes, lectins have a number of biological functions including block to polyspermy at fertilization and defense against pathogens [51]. In the present study, *lcal*, a Ca^2+^-dependent or C-type lectin, was more highly expressed at the CA stage and was the predominant transcript from the CA stage SSH library, which is not surprising given the abundance of CA evident in the ovarian histology. Indeed, C-type lectins are often highly represented in ovary transcriptomes in fish [9,16,52]. In contrast, however, two other CA components, *lrham *and *alv*, were not differentially expressed across stages. Lrham is an oocyte lectin implicated in block of polyspermy that was localized to CA of rainbow trout [53]. Alv is a metalloproteinase first identified in medaka that is released by CA after fertilization to induce zona pellucida (chorion) hardening [54]. Interestingly, when examined as a group, the CA component genes identified in the present study were not transcribed coordinately during oocyte growth as shown in rainbow trout [55].

### Other ovarian transcripts

A subunit of ferritin heavy polypeptide, *fth3*, was the predominant transcript in the P stage library and qPCR verified that mRNA levels were elevated during primary growth. Ferritins are important because they store and transport iron atoms [56]. Since iron is a critical constituent of metalloproteins, such as enzymes and oxygen carriers, it is essential to all organisms. Free iron, however, can be highly toxic to cells and thus ferritin protects cells from the damaging effects of iron, but makes it readily available. Studies have documented high levels of ferritin subunit mRNAs in the ovary [13,49] but little is known about their specific role.

Like ferritin, *dnaja2 *and *ccne *showed significantly higher transcript levels during primary growth. *Dnaja2 *encodes a chaperone protein associated with unfolded protein and heat shock protein binding, while cyclins are positive cell cycle regulators that appear to be profuse in the fish ovary [30,57]. *Ccne *is transcribed and stored during oocyte growth in goldfish and is thought to be important to the first embryonic cell cycles [58]. The high expression of *ccne *during primary growth in this study is consistent with its early transcription in goldfish and suggests it is a classic maternal mRNA, as shown for some other cyclins [27].

*Rdh1 *and *cope *showed an opposite profile with transcript levels higher during early secondary growth. Retinol dehydrogenases are involved in the synthesis of retinoic acid, the active form of vitamin A, which regulates cellular growth and differentiation, embryogenesis, and reproduction in vertebrates [59]. Because of the diverse functions of retinol dehydrogenases, it is unclear what role Rdh1 may play during previtellogenic growth. However, based on the recent detection of other retinoid-related transcripts, such as retinol dehydrogenase type II and retinol binding protein in the trout ovary [11], several players in this cascade are present during oogenesis and likely play an important role during oogenesis and/or early embryogenesis.

The *cope *transcript encodes the epsilon subunit of a coatomer complex protein. One well characterized coatomer protein is clathrin, which is involved in receptor-mediated endocytosis and the transport of proteins from the Golgi network [60]. At this point it is not possible to determine what process the coatomer complex gene identified here is associated with, but perhaps it is involved in uptake of vitellogenin by receptor-mediated endocytosis, protein trafficking, and/or the immune response.

Transcripts for *fshr*, *amh*, *gsdf*, and *lpl*, which are all likely produced in follicle cells, exhibited the largest change in abundance across the P and CA stages with levels increasing 4–5 fold. The increase in *fshr *during this transition from primary to secondary growth together with previous data showing a progressive increase in plasma FSH from the P to lipid droplet stage and effects of FSH on ovarian steroidogenesis [21,61-63], suggest FSH plays an important role in the endocrine control of this phase of oogenesis in coho salmon. It is not known, however, whether the expression of follicle cell transcripts such as those identified in this study is regulated by FSH and/or other endocrine or paracrine factors.

Transcript levels for two members of the TGFβ superfamily, *amh *and *gsdf*, also increased 4–5 fold from the P to CA stage. In female mammals, AMH is produced in the granulosa cells, increases at puberty onset reaching peak levels in small antral follicles, diminishes in later stages, and is no longer detectable during the FSH-dependent final stages of follicle growth or in atretic follicles [64]. Recently, the structure of Amh has been characterized in fish [65,66] and studies have primarily focused on its expression during sex differentiation [39,65]. During oogenesis in zebrafish, *amh *has been localized to granulosa cells where transcript levels peak at the CA stage and progressively decline at onset of yolk incorporation, reaching non-detectable levels by late vitellogenesis. Gsdf is a recently identified gonad-specific cytokine that appears to exist only in teleosts [67]. In rainbow trout, *gsdf *mRNA was localized to somatic cells of the genital ridge during embryogenesis and Sertoli and granulosa cells during gametogenesis [67]. Gsdf plays a role in primordial germ cell and spermatogonial proliferation, but its role in the ovary is unclear, as it did not induce oocyte proliferation in trout. Based on increased *gsdf *transcript levels during secondary growth in coho salmon and the ability of Gsdf to stimulate germ cell proliferation in trout, it is possible this factor plays a role in granulosa cell proliferation that occurs during this period.

## Conclusion

This study sheds light on differentially expressed ovarian genes during previtellogenic oocyte growth in coho salmon and provides a platform for future studies on the regulation of this process. Major gene families represented in the SSH libraries included *zp *genes, lipoprotein receptors, yolk proteases, and CA components, most of which appear to be derived from the oocyte. Transcript abundance was measured for selected genes identified by SSH and candidate genes likely expressed in follicle cells, providing for some genes the first transcriptional profile during early oogenesis in fish. Interestingly, a number of oogenesis related transcripts that will not be utilized until 1–3 years later in coho salmon were highly expressed during primary growth. Clearly, further studies are necessary to determine when these mRNAs are translated and if these transcripts may be subject to masking during early oogenesis. Finally, we observed increased expression of genes encoding FSH receptor, TGFβ family peptides, proteins involved in lipid uptake, and a CA component during the transition from primary to secondary oocyte growth. This period in coho salmon coincides with increased FSH signaling. However, the degree to which FSH regulates differentially expressed genes identified in this study is not known and will be the subject of our future investigations.

## Authors' contributions

JAL participated in the study design, execution of experiments, data analysis and interpretation, and drafted the manuscript. DBI performed the SSH and participated in data analysis. FWG participated in the study design and assisted with data analysis and interpretation. PS participated in the study design, acquired funding, and assisted with data analysis and interpretation. All the authors read, edited, and approved the final manuscript.
